# Efficiency of integrated electrooxidation and anaerobic digestion of waste activated sludge

**DOI:** 10.1186/s13068-021-01929-7

**Published:** 2021-04-01

**Authors:** J. A. Barrios, A. Cano, F. F. Rivera, M. E. Cisneros, U. Durán

**Affiliations:** 1 Instituto de Ingeniería, UNAM, P.O. Box 70-186, 04510 Mexico City, Mexico; 2 CONACYT-Centro de Investigación y Desarrollo Tecnológico en Electroquímica. Parque Tecnológico Querétaro S/N Sanfandila, Pedro Escobedo, Querétaro, C.P. 76703 Mexico, Mexico

**Keywords:** Anaerobic digestion, Waste activated sludge, Pre-treatment, Electrooxidation, Energy analysis

## Abstract

**Background:**

Most of the organic content of waste activated sludge (WAS) comprises microbial cells hard to degrade, which must be pre-treated for energy recovery by anaerobic digestion (AD). Electrooxidation pre-treatment (EOP) with boron-doped diamond (BDD) electrode have been considered a promising novel technology that increase hydrolysis rate, by the disintegrating cell walls from WAS. Although electrochemical oxidation could efficiently solubilize organic substances of macromolecules, limited reports are available on EOP of WAS for improving AD. In this endeavour, the mathematical optimization study and the energy analysis of the effects of initial total solids concentrations [TS] of WAS and current density (CD) during EOP on the methane production and removal of chemical oxygen demand (COD) and volatile solids (VS) were investigated. Because limited reports are available on EOP of WAS for improving biogas production, it is not well understood; however, it has started to attract interest of scientists and engineers.

**Results:**

In the present work, the energy recovery as biogas and WAS conversion were comprehensively affected by CD and [TS], in an integrated EOP and AD system. When working with WAS at 3% of [TS] pre-treated at current density of 24.1 mA/cm^2^, the highest COD and VS removal were achieved, making it possible to obtain the maximum methane (CH_4_) production of 305 N-L/kg VS and a positive energy balance of 1.67 kWh/kg VS. Therefore, the current densities used in BDD electrode are adequate to produce the strong oxidant (hydroxyl radical, ^·^OH) on the electrode surface, allow the oxidation of organic compounds that favours the solubilization of particulate matter and VS from WAS.

**Conclusions:**

The improvement of VS removal and COD solubilization were due to the effects of pre-treatments, which help to break down the microbial cells for faster subsequent degradation; this allows a decomposition reaction that leads to biodegrade more compounds during AD. The balance was positive, suggesting that even without any optimization the energy used as electricity could be recovered from the increased methane production. It is worth noting that this kind of analysis have not been sufficiently studied so far. It is therefore important to understand how operational parameters can influence the pre-treatment and AD performances. The current study highlights that the mathematical optimization and energy analysis can make the whole process more convenient and feasible.
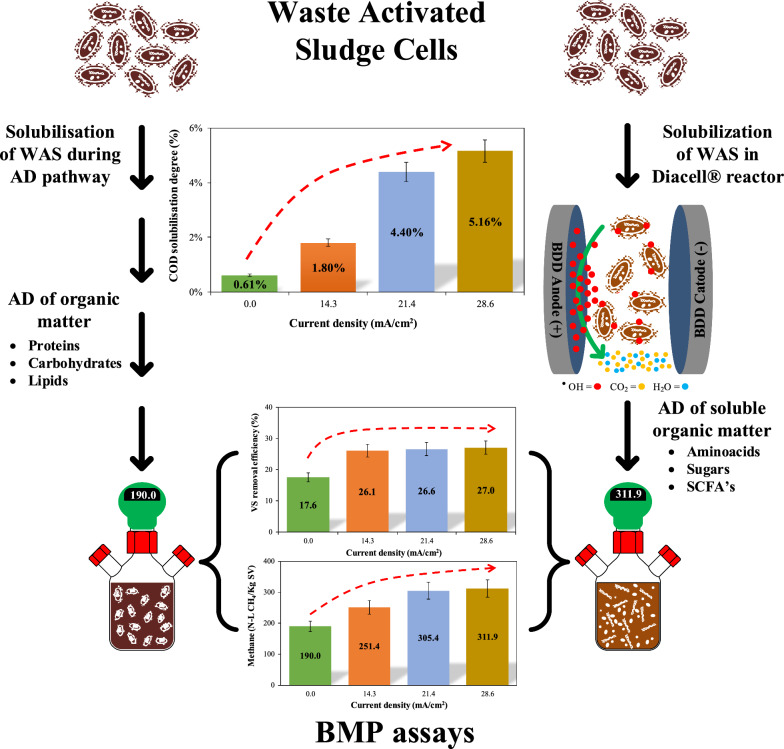

**Supplementary Information:**

The online version contains supplementary material available at 10.1186/s13068-021-01929-7.

## Background

The activated sludge process is currently one the most widely used biological wastewater treatment processes in Latin America, especially for municipal wastewater [1]. In municipal wastewater treatment plant (WWTP), the removal of biodegradable compounds by conventional biological aerobic systems are generating a larger amount of waste activated sludge (WAS). In the last decades, most widely applied practices for sludge disposal are land application (use as a fertilizer in agricultural filed), incineration, disposal in landfill and aerobic (composting) or anaerobic stabilization [2, 3]. However, the contamination of the sludge by pathogens, heavy metals, polycyclic aromatic hydrocarbons, polychlorinated biphenyl or dioxins, limits their harnessing [4]. Therefore, the management of excess WAS (treatment and disposal) is an issue of concern and most challenging task for the wastewater treatment sector.

Additionally, activated sludge WWTPs are fast becoming a high-cost item on municipal budgets amidst the rising electricity tariffs and by increase of the carbon dioxide (CO_2_) indirect emissions being attributed to mainly to higher energy consumption and sludge production [5]. Activated sludge process convert a substantial part (about 50–60%) of the wastewater pollution into sludge, without considering primary suspended solids removal [6]. WAS is the excess biomass from suspended-growth aerobic wastewater treatment systems. Most of the organic content of WAS comprises microbial cells. These cells are hard to degrade as their cell wall and membrane are composed of complex organic materials such as peptidoglycan, teichoic acids, and complex polysaccharides that are not readily biodegradable as they serve as a protective cover to resist osmotic lysis [7, 8]. For these reasons, the use of WAS as renewable source of energy has scarcely been studied at all [9].

One of the most commonly used sludge biological treatment processes is anaerobic digestion (AD); it is estimated that 70% of the sludge are stabilized by this method [10]. This process has a major advantage as biogas is produced, which can be used as an energy source and could play a central role in the interconnected energy infrastructures of the future [11]. However, most of the organic content of WAS comprises microbial cells which significantly reduce the hydrolysis rate [12, 13]. In order to enhance the efficiency of anaerobic digestion of WAS, the rate of hydrolysis needs to be increased applying pre-treatments previously.

A number of different pre-treatment operations and processes have been proposed including biological, chemical, enzymatic, thermal and mechanical [13–15]. WAS pre-treatments offers the following advantages: (a) enhances cell lysis; (b) more bio-available organic matter can be transformed into biogas; (c) the solids mass is further reduced and (d) minimal pollution from unpleasant odours [16, 17]. Most of the pre-treatments to WAS show high potentials to be implemented in an anaerobic digester since they stabilize better the sludge and increase more than 50% the methane produced, reaching 0.31 m^3^ of methane (CH_4_) per kilogram of total sludge eliminated (equivalent to 3.41 kWh). However, no energy assessments are usually considered, because not all the pre-treatment technologies have an energy self-sufficiency to be implemented in WWTPs [18, 19].

Not long ago, the use of sludge EOP has been explored as a field of interest, considering the high oxidation capacity of chemical species formed at different electrode surfaces, for example physisorbed hydroxyl radicals (^·^OH) or homogenous species formation like hypochlorous acid (HClO) [20]. Likely, the following simplified reactions at non-active anodes may take place for the electrooxidation of most organic components in WAS:

1 $$R_{{{\text{WAS}}\;{\text{organic}}\;{\text{compounds}}}} + M\left( {\cdot{\text{OH}}} \right) + H^{ + } + {\text{e}}^{ - } \to M + ROx_{{{\text{solubilized}}\;{\text{COD}}}} + {\text{H}}_{2} {\text{O}}.$$

Electrooxidation process transfer of organic matter from the particulate matter of the WAS to the soluble fraction (facilitating biogas formation). This is because the high capacity of electrochemical hydrolysis is provided by short-lived and energy rich free radicals that carry out disintegrating microbial cell walls. Content of methane and hydrogen sulphide in the biogas depends on the proportion of amino acids and monosaccharides soluble, but in WAS 70–80% of the extracellular organic carbon is in the form of proteins and polysaccharides, hence, this would allow a higher methane and energy content of the biogas [21]. As recently proposed by Pérez-Rodríguez et al*.* [16], hydrolysis rate can be improved if the critical engineering aspects of reactors, such as current density, electrode material, flow and time, in order to reduce the operating inefficiencies of the electrochemical process associated to high-energy consumption.

Although electrochemical oxidation could efficiently decompose the organic substances of macromolecules to smaller ones, limited reports are available on EOP of WAS for improving biogas production. Despite the BDD electrode being widely reported as one of the most stable materials for electrochemical applications [22], there are lack of reports about its application as a pre-treatment for the subsequent AD. In order to reduce the pre-treatment-associated energy consumption, this study proposes a novel approach using electrooxidation with a BDD electrode for improving biogas production through a substantially increase VS removal and COD solubilization in AD. For this purpose, the effect of current density during EOP and the initial total solids concentration on methane (CH_4_) production by AD of WAS assessed. In addition, a mathematical optimization study and the energy analysis of the whole process as a function of these critical parameters (current density and total solids concentration) is presented.

## Results and discussion

### Current density effect of electrooxidation pre-treatment

EOP should be applied at conditions that promote solubilization of organic matter, into low molecular weight compounds, to improve hydrolysis and biodegradability during AD and increase biogas yield. However, this pre-treatment process at current densities higher than 30 mA/cm^2^ may oxidize some organic matter [23]. From experimental factorial design results, WAS with 1 and 2% of [TS], the solubilization of organic matter was proportional to current density. During this process, COD reduction is attributable to direct oxidation through hydroxyl radicals (^·^OH). In contrast, in WAS with 3%, an initial particulate matter reduction was observed, followed by a slight increase. Therefore, higher degree of solubilization values were obtained with 24.1 and 28.6 mA/cm^2^ for 3% [TS], respectively. This is likely attributed to the fact that the current densities used in BDD electrode are adequate to produce the strong oxidant ^·^OH radical on the electrode surface, allowing the oxidation of organic compounds that favours the solubilization of COD from WAS, resulting in an increase of methane production [17, 20]. In addition, formation of homogeneous strong oxidants at the current densities applied, improves the conversion of organic matter into soluble COD, an issue of interest in further analyses [24, 25]. However, these results and conditions had to be further validated by BMP assays.

### VS reduction and COD removal

**Fig. 1:**
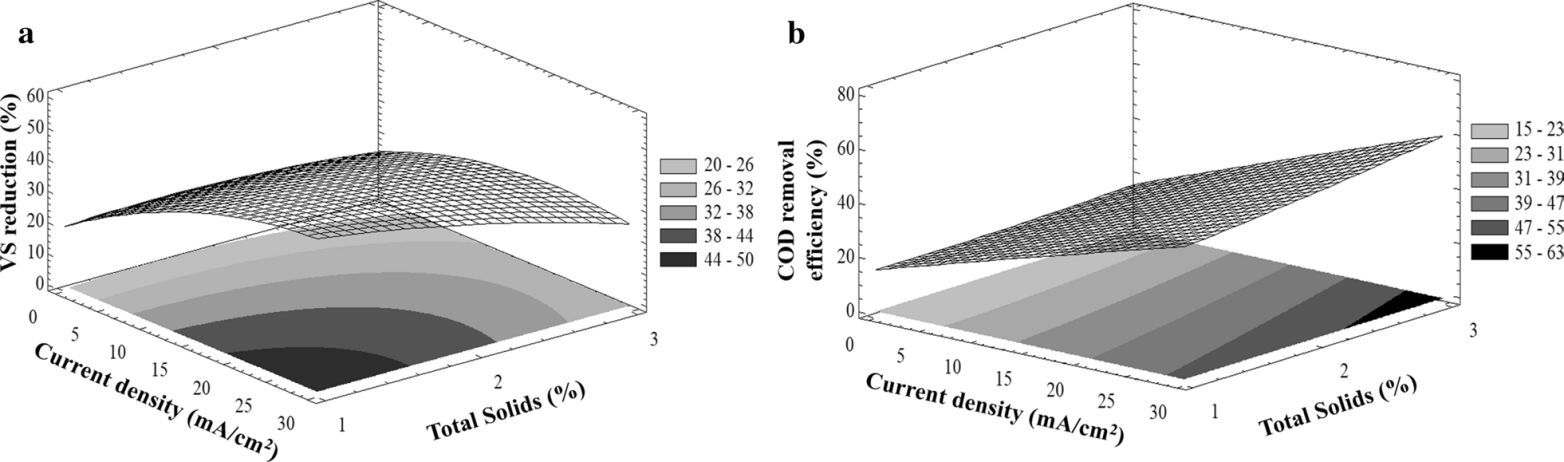
**a** VS reduction and **b** COD removal, in response to different CD and TS

VS removal efficiency of 38% is recommended for the assessment of sewage sludge stabilization, according to standards for the use or disposal of sewage sludge [26]. Removal efficiencies with low TS concentrations (1 and 2%) were higher than 38%. However, at the upper level (3%), these values were lower (Fig. 1a) at the testing conditions and after 16 days. In all cases, VS removals were higher for the electrooxidation pre-treatments, if compared with the 14.2% VS removal obtained with the non-pretreated WAS.

Analysis of variance (ANOVA) shows that the variability of VS removal efficiency (VS_RE_) for each treatment has a *P*-value of < 0.05, indicating that they are significantly different from WAS (confidence level of 95.0%). A data correlation may be obtained with the following fitted model:

2 $${\text{VS}}_{{{\text{RE}}}} \left( \% \right) = 13.95 + 2.17\left[ {{\text{CD}}} \right] + 7.29\left[ {{\text{TS}}} \right] {-}\;0.03[{\text{CD}}]^{2} {-}\;2.28[{\text{TS}}]^{2} - 0.32\left[ {{\text{CD}}} \right]\left[ {{\text{TS}}} \right].$$

Nevertheless, the highest COD removal was achieved with the EOP of 28.6 mA/cm^2^ of current density regardless of TS concentrations, which could be accounted for the disintegration and solubilization of WAS as mentioned above (Fig. 1b). Since it has been reported that the formation of ^·^OH is carried out from 10 mA/cm^2^, the fact of the COD solubilization increase as a function of current density, indicates possible formation of other strong oxidants species, of the kind of RO_2_, in bulk solution increasing the reaction time between solid particles and oxidants [27]. Consequently, if COD solubilization would depend only on the contact between solid particles and physisorbed radicals, a neglected or slight increasing effect on COD solubilization with the current density should be observed [28].

Due to this improvement with an increase of current density, EOP appears to be a promising pre-treatment process. Furthermore, 68% more COD was removed from this compared to the control under the tested conditions (Fig. 1b). Even though VS removal efficiency did not meet the USEPA standard, volatile solids destruction reduces sludge mass that needs to be transported. Nevertheless, these results of Fig. 1 and Eq. (1) allow identifying the time required for producing half of COD soluble and the relative VS removal efficiency for the EOP sludge was identified. In this case, ANOVA analyses shows the variability of COD removal efficiency (COD_RE_) of 2 effects have a *P*-value of < 0.05, indicating that they are significantly different with a confidence level of 95.0%, and from the effects correlation the following fitted model was obtained:

3 $${\text{COD}}_{{{\text{RE}}}} \left( \% \right) = 16.73 + 1.00\left[ {{\text{CD}}} \right] - 2.04\left[ {{\text{TS}}} \right] {-}\;0.001[{\text{CD}}]^{2} + 0.71[{\text{TS}}]^{2} + 0.16\left[ {{\text{CD}}} \right]\left[ {{\text{TS}}} \right].$$

In EOP, current density of 19.3 mA/cm^2^, flow rate of 4 L/min and treatment time of 30 min are required to large molecules contained in sludge particles and microbial cells were partially solubilized, demonstrating that, the process is controlled by mass transfer [22]. The observed result is similar to those reported in the literature [30], where all of them recommended working the electrolysis at current density lower than 30 mA/cm^2^, because might lead the acceleration of organic matter mineralization than the solubilization of WAS. Thus, it is advisable to limit the current density to avoid adverse effects such as heat generation and higher power consumption [31]. Considering this, it was obtained that the electrolysis treatment at current density of 28.6 mA/cm^2^ allowed a fast WAS hydrolysis and the best degree of disintegration.

### Current density effect of the EOP on BMP

A slow biogas generation process was observed in the initial period in all assays, which took around 10 days for 50% total biogas generated. BMP assays of electrooxidized WAS confirmed results obtained from COD solubilization. Electrooxidation pre-treatment enhanced the methane production of WAS from 109 N-L CH_4_/kg VS in non-pretreated WAS to 311.9 ± 6 N-L CH_4_/kg VS with WAS en 3% of TS and EOP to 28.6 mA/cm^2^. This increase of about 203 N-L CH_4_/kg VS (65%), which is more than expected from soluble COD and suggests that the VS disintegration and solubilization resulted from firstly the rapid sludge disintegration during the electrooxidation pre-treatment and then organics available for slow conversion during the anaerobic digestion. Therefore, there exists a positive correlation between the current density in EOP and both methane amount (Eq. 4). In this case, ANOVA analyses show the variability of methane production of treatments have a *P*-value of < 0.05, indicating that they are significantly different with a confidence level of 95.0%. Figure 1 shows a correlation with the following fitted model:

4 $${\text{BMP}}\left( {{ }\frac{{\text{N-L}}}{{\text{kg VS}}}} \right){ = }-{0}{{.99 + 13}}{.79}\left[ {{\text{CD}}} \right]{ + 32}{\text{.78}}\left[ {{\text{TS}}} \right] {-}{0}{\text{.30[CD]}}^{{2}} {-}\;{3}{\text{.66[TS]}}^{{2}} -\;{1}{\text{.38}}\left[ {{\text{CD}}} \right]\left[ {{\text{TS}}} \right].$$

**Fig. 2:**
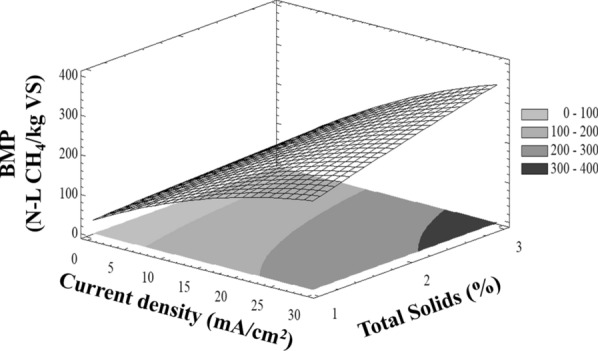
Effect of CD applied and TS on BMP under mesophilic anaerobic digestion

This suggests, as was mentioned before, that other phenomena occur during electrooxidation and they favour solubilization of organic matter which then favours anaerobic digestion. As a result, the improved methane production indicates that the impact of the rate-limiting hydrolysis step could be reduced by electrooxidation pre-treatment. Results in Fig. 2 show that the current density had an impact on the methane production from WAS. The methane production increased proportionally with both TS concentration and current density. This is explained by the fact that the EOP itself (in a single chamber without pH change) could disrupt cell membranes in WAS and therefore enhance biodegradation in subsequent anaerobic digestion [32].

The calculation of maximum BMP as function of CD and TS from Eq. (4) was evaluated using nonlinear complex method [33]. Constrain values employed for this calculation are:

5 $$1\, \le \, [{\rm TS}]\, \le \,{\rm TS}_{\rm op},\, {\rm whit\,TS}_{\rm op} = 1-3.5\%,$$

6 $${\text{CD}}\, \le \,35{\text{ mA/cm}}^{2} .$$

These constraints were established since a low WAS particle dispersion during hydrodynamic tests was observed and the current densities recommend to produce ^·^OH radicals in BDD electrode is < 20 mA/cm^2^ [34]. In this study, current densities greater than 20 mA/cm^2^ were chosen because of the possible hindering of electrode area during particle–electrode interactions [35]. Evaluation of variable TS indicates that the maximum of this equation always occurs at the upper end of such variable as shown in Fig. 1, so the PBM depends directly on the density of applied current, finding the maximum methane production in the extreme values of TS. Having as limiting the difficulty of evaluating the effects of solids concentrations higher than 3.5%, because from the operational point of view it is not possible to work with solids concentrations greater than 3% (Table 1).

**Table 1:** Results of the mathematical maximization of the correlations obtained (Eqs. 2, 3 and 4), with Eqs. (5 and 6) as constrain values

Variables maximized	Initial total solids (%)						
	1.0	2.0	3.0	3.1	3.2	3.4	3.5
Current density (mA/cm^2^)	25.28	27.58	29.88	30.11	30.34	30.8	31.03
BMP_max_ (N-L CH_4_/kg VS)	219.9	278.2	332.3	337.5	342.6	352.8	357.8
COD removal (%)	45.6	50.8	57.6	58.4	59.2	60.8	61.6
VS removal (%)	47.4	39.8	25.9	24.1	22.3	18.5	16.5

### Energy analysis

While methane production was significantly improved through EOP, thermal and electrical energy were also consumed. For industrial application of a suitable pre-treatment, the energy invested in this process should be obtained as an additional methane yield. The energy consumption of the described electrooxidation process can be calculated by Eq. (7) [36, 37]:

7 $$W \left( {\text{kWh/kg VS}} \right){ = }\frac{{{\text{(V}} \times {\text{A}} \times {\text{t)/1000}}}}{{\text{[VS]}}},$$

where *V* is the average supplied voltage in W/A, *A* is the amps, *t* is the operation time in hours and [VS] is initial volatile solids mass in kg.

Under the best conditions, the energy consumption of the EOP was 1.07 kWh/kg VS, suggesting that even without any optimization the energy used as electricity could be approximately recovered as energy from the increased methane production (305 N-L CH_4_/kg VS), which can produce about 3.43 kWh/kg VS. A summary of performance and energy outcomes for the major pre-treatments and options is given in Table 2. This is information standardized from the various sources based on solids concentration as kg VS. A nominal VS:TS ratio of 84% was used. Calorific values and heat capacities have been taken from standard texts [37]. In general, where a range of performance measures has been used, the more widely industrially applied examples, or best conditions have been applied [36]. Considering the electrical and thermal available energy, for the sludge pre-treated by EOP, the cogeneration would produce approx. 0.59 kWh/kg VS as electricity and 0.84 kWh/kg VS as heat, respectively (Additional file 1: Sect. 2.1). All options for pretreatment have substantial capital cost, however, with EOP being more capitally intensive than other options [17, 29]. Often, the energy balance by other extras exceed substantially the energy use of the actual pre-treatment equipment [38]. However, the energy analysis of the present study only considers the main pre-treatment unit operations.

**Table 2:** Energy analysis of EOP with mesophilic anaerobic digestion compared to other pre-treatment methods, modified from Barrios et al. [20], Cano et al. [32] and Carrère et al. [31]

Pre-treatment method (conditions)	Energy consumption^a^				BMP^c^, in L-N CH_4_ (kWh) per kg VS
	Pre-treatment (kWh/kg VS)	Electricity^b^ (kWh/kg VS)	Thermal energy^b^ (kWh/kg VS)	Total, (kWh/kg VS)	
Non-pretreatment	–	0.29	0.50	0.79	190 (2.09)
Thermal hydrolysis (170 °C for 15–30 min)	0.94	0.29	0.50	1.73	291 (3.20)
Sonication (100 W, 16 s, 30 kW m^−3^)	0.37	0.29	0.50	1.16	241 (2.65)
Ball milling	1.04	0.29	0.50	1.83	241 (2.65)
High pressure (200 bar)	0.33	0.29	0.50	1.12	261 (2.87)
Present study, EOP at: 14.3 A m^−2^, 30 min	0.25	0.29	0.50	1.03	251 (2.76)
21.4 A m^−2^, 30 min	0.38	0.29	0.50	1.17	305 (3.36)
28.6 A m^−2^, 30 min	0.51	0.29	0.50	1.30	312 (3.43)

^a^Analysis assumes a hydraulic retention time of 16 days for mesophilic anaerobic digestion and the energy consumption as pre-treatment, electricity and thermal energy is considered

^b^Details on electrical and thermal energy calculations are in section (a) of the Additional file 1, and details on energy consumption by the EOP is provided in the Additional file 2

^c^A simple mathematical correlation representing a calorific value of 11 kWh N-m^−3^ CH_4_ was set, this value was obtained from the conversion of the methane energy molar in temperature (0 °C) and atmospheric pressure (1 atm) standard conditions, section (b) of the Additional file 1

We have therefore a variation of COD and VS removal after anaerobic digestion, and each pre-treatment gave an advantage in COD removal improvement compared to un-pretreated sludge. The highest COD and VS removal were achieved with sludge pre-treated at 3% of TS and current density of 24.1 mA/cm^2^, and thus the maximum biogas production was achieved. The improvement of VS removal was due to the effects of pre-treatments, which help to break down the microbial cells for faster subsequent degradation; this allows a decomposition reaction that leads to biodegrade more compounds during anaerobic digestion.

### Overall understanding and implications

Mechanism of electrooxidation pre-treatment enhancing anaerobic digestion of WAS was firstly investigated. It was found that the electrooxidation at 24.1 mA/cm^2^ showed the highest COD and VS removal and it was possible to obtain the maximum methane (CH_4_) production of 305 N-L/kg VS and a positive energy balance of 1.67 kWh/kg VS. Therefore, EOP could remarkably enhance the solubilization of particulate matter and VS from WAS, offering more bio-available organics for methane-producing microorganisms. Obtained results can be employed to conservatively evaluate the technical- and environmental-feasibility of an integrated EOP and AD system. However, semi-continuous operation of the suggested approach needs to be conducted to further evaluate the impact of EOP on WAS anaerobic digestion in real conditions.

## Conclusions

The application of electrooxidation pre-treatment at different TS concentrations was carried out for improving WAS anaerobic digestion. The effectiveness of this method was compared to un-pretreated WAS. The highest COD and VS removal were achieved with sludge pre-treated at 3% of TS and current density of 21.4 mA/cm^2^. The maximization of biogas production indicates that the maximum degradation and methane production depends directly on the applied current density. This study shows a high prospective of an integrated EOP and AD system, because produced 305 N-L CH_4_/kg VS and a positive energy balance of 1.67 kWh/kg VS. Therefore, the results can be employed to conservatively evaluate the technical- and environmental-feasibility of similar systems.

## Methods

The experimental work was focused on BMP assays from pre-treated WAS. These were compared with results of un-pretreated WAS used as reference.

### Sludge samples

Samples of WAS were collected from the Cerro de la Estrella wastewater treatment plant (WWTP), Iztapalapa, Mexico City. This facility treats 2300 L/s of municipal sewage using a conventional activated sludge process. WAS concentrations being as follow: TS (80 ± 4.3 g/L), VS/TS fraction (59.5 ± 11.4%), total chemical oxygen demand (COD) (23.0 ± 6.1 g/L), soluble COD (2.4 ± 0.9 g/L), carbohydrates (29.0 ± 5.3% of VS), protein (4.3 ± 3.2% of VS) and oil and grease (1.0 ± 0.9% of VS).

### Inoculum source

Anaerobic sludge used as inoculum for BMP assays was collected from a brewery WWTP. This sludge was initially used for the start-up of a seed digester fed with un-pretreated WAS. Once the seed reactor reached steady state, the resulting sludge was used as inoculum for BMP assays. To avoid interference of biogas production from the remnant substrate, the anaerobic sludge inoculum was incubated for 24 h in a vacuum chamber before seeding the BMP bottles.

### Electrooxidation pre-treatment (EOP)

A Diaclean^®^ electrochemical reactor composed by two circular electrodes and two spacers was used for the experiments. The assays were carried out in a single-compartment electrochemical reactor. Diamond-based material (*p*-Si–BDD) was used as anode and cathode. Both electrodes were circular (100 mm diameter) with a surface area of 70 cm^2^. The relevant dimensions of the electrochemical reactor are similar to the reported by Barrios et al*.* [35]. The electrochemical reactor was coupled to a hydraulic system consisting of a 4-L reservoir made of glass and a peristaltic pump (JP Selecta Per-com N-M328). Tubes, valves, and accessories were made of PVC. Sludge was stirred in the glass reservoir with an overhead mixer (stainless steel paddle area: 49 cm^2^) to avoid solids settling. The stirrer speed was low (100 rpm) in order to keep the sludge homogeneous and avoid phase separation at the reactor entrance. Power was supplied by a Delta Elektronika ES030-10, applying current densities of 14.3, 21.4 and 28.6 mA/cm^2^ during 30 min. The temperature in the reservoir was kept constant (25 °C) with a water bath system.

### Biochemical methane potential assays

The anaerobic digestion for un-pretreated (pre-treatment control) and pre-treated WAS was measured in an OxiTop^®^ Control OC 110. BMP assays were performed with a working volume of 80 mL, in 250-mL flasks and the increase of pressure inside the headspace were stored in the OxiTop measuring head at every day intervals automatically. BMP assays were carried out at mesophilic temperature (36 ± 2 °C) during 16 days, initial pH was adjusted to seven and flasks shaken at 150 rpm. The amount of WAS and inoculum were calculated using a substrate/initial biomass (*S*/*X*_0_) ratio of 0.5 g VS_fed_/g VS_biomass_. Optimization of the selected operating conditions was assessed by the response surface methodology. A 3-level full factorial design was performed (Table 3), the factors were the WAS concentration as total solids [1.0, 2.0 and 3.0% (w/v)] and the current density of the EOP (0 as control, 14.3, 21.4 and 28.6 mA/cm^2^). The influence of treatments was separated into the main effects of total sludge concentration versus current densities and the interaction between these two factors. The controls used were: (a) a negative control (inoculum without substrate) to determine the endogenous production of CH_4_, and (b) a bottle with water at the same volume to correct pressure measurements of the system.

**Table 3:** Full factorial design with [TS] as A and [CD] as B factors, with three levels for both parameters

[TS] (%)	Current density (mA/cm^2^)		
	[CD]_1_ (14.3)	[CD]_2_ (21.4)	[CD]_3_ (28.6)
[TS]_1_ (1.0)	[TS]_1_, [CD]_1_	[TS]_1_, [CD]_2_	[TS]_1_, [CD]_3_
[TS]_2_ (2.0)	[TS]_2_, [CD]_1_	[TS]_2_, [CD]_2_	[TS]_2_, [CD]_3_
[TS]_3_ (3.0)	[TS]_3_, [CD]_1_	[TS]_3_, [CD]_2_	[TS]_3_, [CD]_3_

To standardize the BMP results, methane produced was expressed in terms of the normalized litre (N-L), gas volume must be converted to standard conditions (0 °C at 1 atm).

### Analytical methods

Total solids (TS), volatile solids (VS), fixed solids (FS), pH, total alkalinity, and soluble chemical oxygen demand (COD was analysed after a 1:20 dilution of sludge samples) were determined according to the Standard Methods [39]. Alkalinity ratio, α, was determined by dividing the partial alkalinity (pH 5.75) and the total alkalinity (pH 4.3). The concentration of volatile fatty acids (VFA) was measured by gas chromatography (SRI 8610-10 with flame ionization detector, N_2_ as carrier gas using an Alltech EC-1000 column). Biogas volume was determined by the OxiTop^®^ system, while biogas composition was analysed by gas chromatography (FISHER Gas Partitioner chromatograph model 1200) with thermal conductivity detector, He as carrier gas and a Porapak Q column.

### Maximization of methane production

From the experimental data obtained at different [CD] and [TS] values, the removal of COD and VS and the methane production were theoretically maximized through a mathematical optimization analysis. To maximize the expressions obtained, a nonlinear complex method was used. Once the objective function is determined, its derivatives are calculated and the critical point (where objective function is maximized) is obtained by means of Hessian matrices method. This methodology was implemented in Excel and mathematical details of this procedure are shown in “Results” section. The purpose of this data treatment was to determine the conditions associated with the biogas maximum production (Additional file 2).

### Statistical analysis

Statistical analysis of the BMP assays results was carried out using STATGRAPHICS Centurion XVI version 16.1.03 software. The ANOVA test was implemented to evaluate if differences could be observed between the different current densities for each sludge concentration, after which post hoc multiple comparison was carried out by means of the Tukey HSD test at the 5% significance level. In all BMP assays, methane yields were reported as the average of replicate samples, and reported as mean ± standard deviation.

## Supplementary Information

**Additional file 1** EOP-AD system energy production and consumption calculations.

**Additional file 2** Energy consumption calculations by EOP.

## Data Availability

The datasets used and/or analysed during the current study are available from the corresponding author on reasonable request.

## Abbreviations

AD: Anaerobic digestion
ANOVA: Analysis of variance
BDD: Boron-doped diamond
BMP: Biochemical methane potential
CD: Current density
COD: Chemical oxygen demand
COD_RE_: COD removal efficiency
CO_2_: Carbon dioxide
EOP: Electrooxidation pre-treatment
HClO: Hypochlorous acid
^·^OH: Hydroxyl radicals
N-L CH_4_: Normalized methane
*S*/*X*_0_: Substrate/initial biomass ratio
TS: Total solids concentration
VFA: Volatile fatty acids
VS: Volatile solids
VS_RE_: VS removal efficiency
WAS: Waste activated sludge
WWTP: Wastewater treatment plant
