# Supplementary material for: Efficiency of integrated electrooxidation and anaerobic digestion of waste activated sludge
Source: Biotechnol Biofuels. 2021 Apr 1;14:81. doi: 10.1186/s13068-021-01929-7 (PMC8017613; doi:10.1186/s13068-021-01929-7)
Supplement: Supplementary file 1 — Additional file 1 EOP-AD system energy production and consumption calculations. [file 13068_2021_1929_MOESM1_ESM.docx]

**Efficiency of an integrated process of electrooxidation and anaerobic digestion of waste activated sludge**

J.A. Barrios^a^, A. Cano^a^, F.F. Rivera^b^, M.E. Cisneros^a^, U. Durán^a*^

* Corresponding author. (E-mail: [UDuranH@iingen.unam.mx](mailto:UDuranH@iingen.unam.mx))

Additional file 1

# RESULTS AND DISCUSSION

## Energy consumed of EOP with mesophilic anaerobic digestion compared to other pre-treatment methods.

The analysis assumes a 16-day period for mesophilic anaerobic digestion at 37 ° C. The energy consumption is electrical (20% by pumping and 80% by mixing) and thermal. A concentration of volatile solids of 8 kg/m^3^ was used as the basis for calculation. The electrical and thermal requirements for this analysis are [1,2]:

- Pumping = 0.12 kW-h/m^3^/d = 0.015 kW-h/kg VS/d
- Mixed = 0.12 kW-h/m^3^/d = 0.015 kW-h/kg VS/d
- Heating = 0.25 kW-h/m^3^/d = 0.031 kW-h/kg VS/d

| **Pre-treatment method*** | **Conditions** | **Energy consumption** | | | | | **BMP (N-L CH_4_/kg VS)** | **BMP (kW-h/kg VS)** | **Energy balance**  **(kW-h/kg VS)** |
| --- | --- | --- | --- | --- | --- | --- | --- | --- | --- |
|  |  | **Electrical energy by pre-treatment (KW-h/Kg VS)** | **Electrical energy by pumping (KW-h/Kg VS)** | **Electrical energy by mixing (KW-h/Kg VS)** | **Thermal energy by heating (KW-h/Kg VS)** | **Total (KW-h/Kg VS)** |  |  |  |
| Unpre-treatment | - | 0.000 | 0.096 | 0.192 | 0.500 | 0.788 | 190 | 2.09 | 1.30 |
| Thermal hydrolysis | 170 °C for 15–30 min | 0.938 | 0.096 | 0.192 | 0.500 | 1.726 | 291 | 3.20 | 1.47 |
| Sonication | 100 W, 16 s, 30 kW/m^3^ | 0.370 | 0.096 | 0.192 | 0.500 | 1.158 | 241 | 2.65 | 1.49 |
| Ball milling | - | 1.040 | 0.096 | 0.192 | 0.500 | 1.828 | 241 | 2.65 | 0.82 |
| High pressure | 200 bar | 0.330 | 0.096 | 0.192 | 0.500 | 1.118 | 261 | 2.87 | 1.75 |
| Present study with EOP | 14.3 A/m^2^, 30 min | 0.247 | 0.096 | 0.192 | 0.500 | 1.035 | 251 | 2.77 | 1.73 |
|  | 21.4 A/m^2^, 30 min | 0.377 | 0.096 | 0.192 | 0.500 | 1.165 | 305 | 3.36 | 2.19 |
|  | 28.6 A/m^2^, 30 min | 0.512 | 0.096 | 0.192 | 0.500 | 1.300 | 312 | 3.43 | 2.13 |
| Conditions obtained by Carrere *et al*. [3] | | | | | | | | | |

## Normalized energy produced by assays of EOP with mesophilic anaerobic digestion

CALCULATIONS:

CH_4_ + 2O_2_ → CO_2_ + 2H_2_O (ΔG = 212 kCal/mol)

1 mol = 24.4 L (a 0 °C)

MW_CH4_ = 16 g/mol

1 m^3^ = 40.98 mol Conversion by ideal gases equation

212 kCal/mol x 44.64 mol/m^3^ =

As such: 1 kW-h = 860 kCal

9,463.68/860 = 11.00 kW-h

Therefore, 1 m^3^ of CH_4_ has an energy of 11.00 kW-h (0 ° C), according to Cano et al. (2015)

In general, efficiency is affected when converting, so it is usually used for 35% electric power and 50% for thermal energy.

Electric energy of 1 m^3^ of CH_4_ = 11.0 x 0.35 = 3.85 kW-h

Thermal energy of 1 m^3^ of CH_4_ = 11.0 x 0.50 = 5.50 kW-h

**Energy produced in assays**

| **Assays** | **CH_4_ (N-L/Kg SV_initials_)** | | | **Energy (kW-h)** | | | **Electric energy (kW-h)** | | | **Thermal energy (kW-h)** | | |
| --- | --- | --- | --- | --- | --- | --- | --- | --- | --- | --- | --- | --- |
|  | **1%** | **2%** | **3%** | **1%** | **2%** | **3%** | **1%** | **2%** | **3%** | **1%** | **2%** | **3%** |
| **0.0** | 52 | 47 | 37 | 0.57 | 0.51 | 0.40 | 0.20 | 0.18 | 0.14 | 0.29 | 0.26 | 0.20 |
| **14.3** | 130 | 175 | 251 | 1.42 | 1.93 | 2.77 | 0.50 | 0.67 | 0.97 | 0.71 | 0.96 | 1.38 |
| **21.4** | 180 | 258 | 305 | 1.98 | 2.84 | 3.36 | 0.69 | 0.99 | 1.18 | 0.99 | 1.42 | 1.68 |
| **28.6** | 240 | 288 | 312 | 2.65 | 3.17 | 3.43 | 0.93 | 1.11 | 1.20 | 1.32 | 1.59 | 1.72 |
|  |  |  |  |  |  |  |  |  |  |  |  |  |

## REFERENCES

1. Barrios JA, Duran U, Cano A, Cisneros-Ortiz M, Hernández S. Sludge electrooxidation as pre-treatment for anaerobic digestion. Water Sci Technol. 2017;75:775–81.

2. Pérez-Rodríguez M, Cano A, Durán U, Barrios JA. Solubilization of organic matter by electrochemical treatment of sludge: Influence of operating conditions. J Environ Manage. 2019;236:317–22.

3. Carrère H, Dumas C, Battimelli a, Batstone DJ, Delgenès JP, Steyer JP, et al. Pretreatment methods to improve sludge anaerobic degradability: a review. J Hazard Mater. 2010;183:1–15.

4. Cano R, Pérez-Elvira SI, Fdz-Polanco F. Energy feasibility study of sludge pretreatments: A review. Appl Energy [Internet]. Elsevier Ltd; 2015;149:176–85. Available from: http://dx.doi.org/10.1016/j.apenergy.2015.03.132
